# Sleep Is a Refreshing Process: An fNIRS Study

**DOI:** 10.3389/fnhum.2019.00160

**Published:** 2019-05-14

**Authors:** Adile Oniz, Gonca Inanc, Serhat Taslica, Cagdas Guducu, Murat Ozgoren

**Affiliations:** ^1^Department of Biophysics, Faculty of Medicine, Dokuz Eylul University, Izmir, Turkey; ^2^Sleep and Conscious States Technology Research and Application Center, Izmir, Turkey; ^3^Department of Biophysics, Institute of Health Sciences, Dokuz Eylul University, Izmir, Turkey; ^4^Faculty of Medicine, Near East University, Nicosia, Cyprus

**Keywords:** sleep, fNIRS, prefrontal cortex, refreshment, brain hemodynamics

## Abstract

Sleep is a very critical process that constitutes up to one third of daytime of a healthy adult. It is known to be an active period where body and brain is refreshed for the next day. It is both part of a larger cycle, i.e., circadian rhythm, and has subcsycles in it, i.e., sleep stages. Although hemodynamics of these stages have been investigated especially in the last two decades, there are still points in the hemodynamics to be illuminated especially in terms of refreshment. This study aims to investigate refreshing property of sleep in terms of sleep stages using functional near-infrared spectroscopy (fNIRS) for measuring prefrontal cortex (PFC) hemodynamics. Nine healthy subjects slept in sleep laboratories, monitored by polysomnography and fNIRS before, during, and after night sleep. REM stage had lower oxyhemoglobin (HbO) and total hemoglobin (HbT) than the other sleep stages and wakefulness. Deoxyhemoglobin (HbR) did not differ between any stages. All sleep stages and wakefulness stage at the end of the sleep had higher HbO and lower HbR than the beginning of the sleep. HbT levels did not differ between the beginning and the end of the sleep for any stages. During REM sleep, PFC seems to get lower blood supply, possibly due to increased demand in other brain regions. Regardless of the stage, PFC has higher oxygenation toward the end of sleep, indicating refreshment. Overall, our brain seems to be on duty during sleep throughout the night for “cleaning” and “refreshing” itself. Hemodynamic changes from the beginning to end of sleep might be the indicator of this work. Thus, accordingly REM stage seems to be at a central point for this work.

## Introduction

Sleep is considered as a very critical process for the physical health as well as mental health. A healthy person spends approximately one third of their lives during sleep. Sleep is the prominent change in brain function dynamics during circadian rhythm. Sleep consists of two basic stages (Iber et al., 2007). One of them is the stages with non-rapid eye movement (NREM), and the other is the rapid eye movement (REM). The NREM may further be divided into three sub-stages: N1, N2, and N3. During regular sleep humans dynamically shift from light to deep sleep and REM stages. Polysomnography systems (PSGs) are used for recording and analysis of various different physiological variables during sleep. The basic recording components of PSG are electroencephalography (EEG), electrooculography (EOG), and electromyography (EMG) which are used to determine the sleep stages.

However, the neurophysiological activities of the brain during sleep still remains as one of the lesser known and key topics in neuroscience. In addition to the neurophysiology,brain hemodynamic activities during the sleep are still unclear or not well-defined. In the last decade, a number of signal processing techniques were applied to determine the sleep stages automatically. Measuring hemodynamic signals seems to be one of the candidates for the future technologies. There are certain studies in the literature, which aimed to clarify the sleep process in the aspect of neurophysiology (Harris, 2005; Schwartz and Roth, 2008) and hemodynamic changes (Näsi et al., 2011; Klingelhöfer, 2012). With the advanced development of the technology, various neuroimaging techniques have been flourished in the field of investigating the brain hemodynamic responses. In the sleep related studies, the characteristics and possible markers of stage transitions have been planned to be explored by the various imaging techniques; such as Positron emission tomography (PET), functional magnetic resonance imaging (fMRI), and functional near-infrared spectroscopy (fNIRS) (Braun et al., 1997; Kjaer et al., 2002; Kaufmann et al., 2006; Horovitz et al., 2008; Laffan et al., 2010). For example, glucose metabolism is observed to be highly decreased during deepest stages of the sleep that has very low frequency oscillations (Dang-Vu et al., 2005). However, this is interpreted as a specific synchronized high activity in deep sleep rather than overall tranquility (Maquet, 2010).

On the other hand, PET and fMRI are not suitable to examine cerebral hemodynamic changes in sleep measurements whole night, as the participants must lie still. Due to their size and operating principles, fMRI systems are not designed for mobility. In addition, fMRI has high initial and running costs. In contrast; fNIRS emerges as a more accessible method because of the mobility and low cost.

In the literature, there is a high correlation between fNIRS and fMRI methods, and fNIRS can reliably be used -instead of fMRI- for a number of research topics (Strangman et al., 2002). Accordingly, fNIRS has become a promising tool and it has also appeared in sleep research area in recent years.

In literature, several fNIRS studies have been conducted to investigate brain hemodynamics changes between sleep stages during sleep in healthy participants. Even, brain and muscle hemodynamics were compared in regard to different stages by fNIRS (Zhang and Khatami, 2015). In one study the oxygen saturation of the brain tissue after sleep was compared with the one prior to sleep (Metz et al., 2013). But there has been no comparison of brain oxygenation values of sleep stages between the beginning and end of sleep.

Accordingly, the current study aims to investigate hemodynamic changes in time course together with sleep stages in order to discuss brain’s work on itself throughout the night sleep. We hypothesized that the brain would refresh itself or replenish energy stock manifested by increasing the oxygenation at the end of the sleep and this would be performed mostly in REM sleep.

## Materials and Methods

Nine healthy subjects [two males, age (mean ± standart deviation): 20.56 ± 1.42 years], have participated in the study. The research has been conducted in Sleep Dynamics Research Laboratories of Department of Biophysics in Dokuz Eylul University (SDRL-DEU). This study was carried out in accordance with the recommendations of Dokuz Eylul University Non-invasive Research Ethics Board with written informed consent from all subjects. All subjects gave written informed consent in accordance with the Declaration of Helsinki. The protocol was approved by the Non-invasive Research Ethics Board. Each participant slept alone at the laboratory for one night. Therefore, the data in this research only covers their first night sleep. But in order to make the participants familiarize with the recording environment and reduce first night effect, they spent around 2 h in the recording room before starting the data acquisition.

Before starting the recording, subjects filled out Edinburgh Handedness Inventory, SCL-90R, STAI Form TX-1, Pittsburgh Sleep Quality Index (PSQI) and Epworth Sleepiness Scale. None of the subjects had any psychiatric, neurological, chronic disease, or sleep problems. All of the subjects had right hand dominance.

The sleep recording has been performed in an electromagnetically isolated room. The room had also sound isolation in order to create extremely silent environment and was dimly lit during the recordings. All subjects were recorded by video camera during sleep.

This research study utilized both Polysomnography (electroencephalography) and hemodynamics recordings during full night (Recording durations are given in Table 1).

**Table 1:** Time spent by participant in bed and during sleep (in total and by stages).

	Duration (h) mean ± SD
In bed	7.06 ± 1.10
Total sleep	6.58 ± 1.05
W	0.48 ± 0.19
N1	0.72 ± 0.39
N2	2.99 ± 0.37
N3	2.00 ± 0.42
REM	0.87 ± 0.35


*W, wakefulness; N1, stage 1; N2, stage 2; N3, stage 3; REM, rapid eye movement stage.*

Polysomnography (PSG) was recorded via NuAmps 40 channel amplifier (Neuro-scan Labs, United States). Inion-nasion distances of each participant were measured in order to place EEG cap on exact 10–20 system positions (Jasper, 1958). The conductivity of the electrodes embedded in the cap was ensured by using conductive gel (ECI Electro-Gel, ElectroCap International, Inc., United States). EEG electrodes were referenced to linked electrodes on the earlobes [(A1+A2)/2]. The earlobes were cleaned with an abrasive skin prepping gel (NuPrep; United States) and ethanol. Reference electrodes were filled with EEG paste (EEG Paste Z-401 CE, Elefix Nihon-Kohden; United States). Electrooculography (EOG) recordings were obtained by placing the electrodes to the outer canthus of each eye. Electromyography (EMG) recordings were made by placing the electrodes to upper and lower sides of the chin. During the recording, impedances of the electrodes were maintained around 10 kOhm.

**FIGURE 1:**
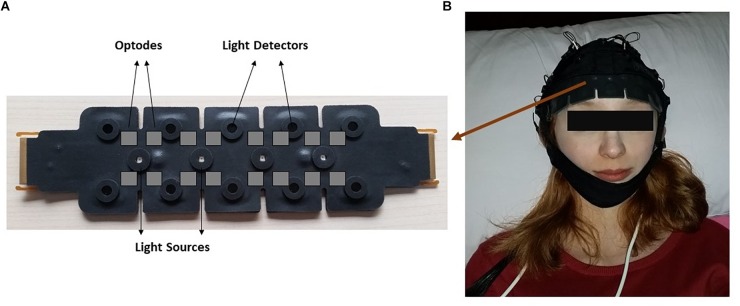
The demonstration of functional near infrared spectroscopy (fNIRS) sensor pad. **(A)** Light sources and detectors on the sensor pad is displayed. Gray squares indicate imagery optode region. **(B)** Localization of the sensor pad on a participant’s forehead.

The measurements were taken from the forehead regions of the participants by the fNIR system (Imager 100, fNIR Devices LLC, Potomac, MD, United States). This system is connected to a flexible sensor pad. The sensor pad contains four light sources with wavelengths of 730 and 850 nm and 10 detectors (Figure 1). The forehead area was cleaned with alcohol swab and scrubbing cream, then the sensor was placed to the forehead region.

Functional near-infrared spectroscopy acquisition was conducted using COBI Studio software (Ayaz et al., 2012). This device calculates relative changes to baseline values of oxy-hemoglobin (HbO), deoxyhemoglobin (HbR) and total hemoglobin (HbT) molecules by means of a continuous wave spectroscopy system that applies light to tissue at constant amplitude. Modified Beer-Lambert Law is applied for the calculations (Baker et al., 2014).

PSG and fNIR analysis have been conducted offline. Sleep recordings were scored according to American Academy of Sleep Medicine (AASM) scoring system. EEG, EMG, and EOG epochs of 30 s were analyzed one-by-one by means of an experienced operator. Single sleep stage was determined for each epoch. If less than 50% of the EEG frequency consisted of alpha rhythm and there was mixed frequency activity with low amplitude in the epoch, it was named stage 1. If the alpha rhythms were not seen clearly in the epoch with predominant 4–7 Hz activity, vertex sharp wave or slow eye movements were seen, the epoch was evaluated as stage 1 again. If there was one or more arousal unrelated K complex or sleep spindle in the first half or in the second half of the previous epoch, it was named stage 2 and continued to be called stage 2 until wakefulness, arousal, stage 3 or REM transition were observed. If slow wave activities with a frequency of 0.5–2 Hz and end-to-end amplitude greater than 75 μV were observed in 20% or more of the epoch, it was named stage 3. If there was low amplitude, mixed frequency EEG waves, REMs, and decreased muscle tone were seen in the epoch, it was defined as REM.

In fNIRSoft standard edition software (Biopac, United States), filtering was performed with a FIR (finite impulse response) low-pass filter to remove the cardiac noise or other biological and movement-related artifacts. For the sake of simplicity in the text, wakefulness data including prior-to-sleep, awakenings throughout the night, and post-sleep are named as wakefulness stage from now on. Mean values for ΔHbO, ΔHbR, and ΔHbT for every occurrence of wakefulness (W), N1, N2, N3, and REM stages were evaluated.

In order to analyze hemoglobin level differences between different stages (i.e., four sleep stages and wakefulness stage), hemoglobin data from each occurrence of any stage is used as dependent variable and stage is set as grouping factor in one-way ANOVA with Tukey’s *post hoc* test. Hence if the subject had seven separate REM stages throughout the night, mean value for each REM stage was used separately for the analysis. Every mean value for each occurrence of the stage for nine subjects is included in the analysis. In order to analyze effect of time on hemoglobin level and its interaction with stages, two-way repeated measures ANOVA was performed with Bonferroni correction for *post hoc* tests. In this analysis, mean values from first and last occurrence of each specific stage was used. Mauchly’s Test of Sphericity indicated that the assumption of sphericity had not been violated in any case. Descriptive values are given as “observed mean ± standard error” for one-way ANOVA results and as “estimated marginal mean ± standard error” for repeated measures ANOVA results. All statistical analyses were performed using IBM SPSS Statistics 24.

## Results

### Sleep Stages

#### ΔHbO

**FIGURE 2:**
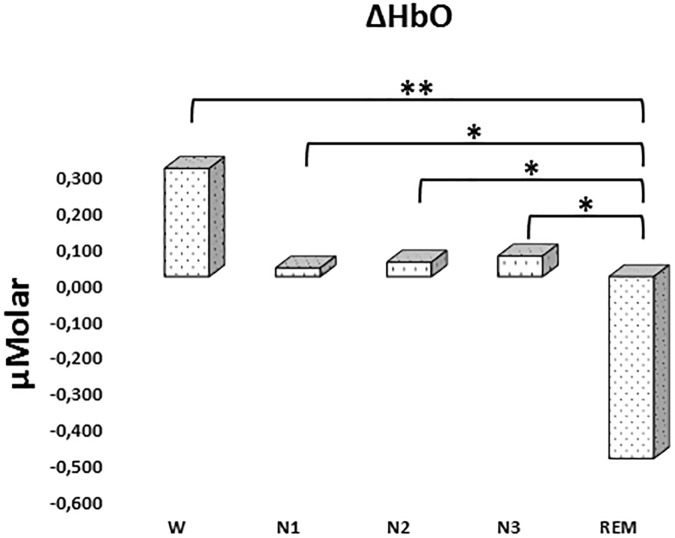
Mean ΔHbO levels for each stage throughout the night. Vertical axis represents oxyhemoglobin level compared to baseline fNIRS measurement in μMolar. Each bar represents separate sleep stage. W, wakefulness; N1, stage 1; N2, stage 2; N3, stage 3; REM, rapid eye movement stage (significant differences are denoted as follows: ^∗^*p* < 0.05, ^∗∗^*p* ≤ 0.01).

There was a statistically significant difference between ΔHbO levels in different stages as determined by one-way ANOVA [*F*(4,907) = 3.418, *p* = 0.009]. A Bonferroni *post hoc* test revealed that ΔHbO was statistically significantly lower in REM (-0.592 ± 0.187) compared to W (0.299 ± 0.130, *p* = 0.002), N1 (0.024 ± 0.097, *p* = 0.048), N2 (0.040 ± 0.087, *p* = 0.028), and N3 (0.057 ± 0.101, *p* = 0.032) (Figure 2). There was no statistically significant difference in other comparisons (*p* > 0.480) (Table 2).

**Table 2:** Hemoglobin levels (observed means ± standard error) compared to baseline recording for each stage throughout the night.

	*ΔHbO (μMolar)*	*ΔHbR (μMolar)*	*ΔHbT (μMolar)*
*W*	0.299 ± 0.130	0.135 ± 0.087	0.435 ± 0.158
*N1*	0.024 ± 0.097	0.287 ± 0.073	0.310 ± 0.143
*N2*	0.040 ± 0.087	0.262 ± 0.061	0.301 ± 0.128
*N3*	0.057 ± 0.101	0.207 ± 0.076	0.265 ± 0.154
*REM*	-0.592 ± 0.187	-0.037 ± 0.131	-0.698 ± 0.275


*W, wakefulness; N1, stage 1; N2, stage 2; N3, stage 3; REM, rapid eye movement stage.*

#### ΔHbR

**FIGURE 3:**
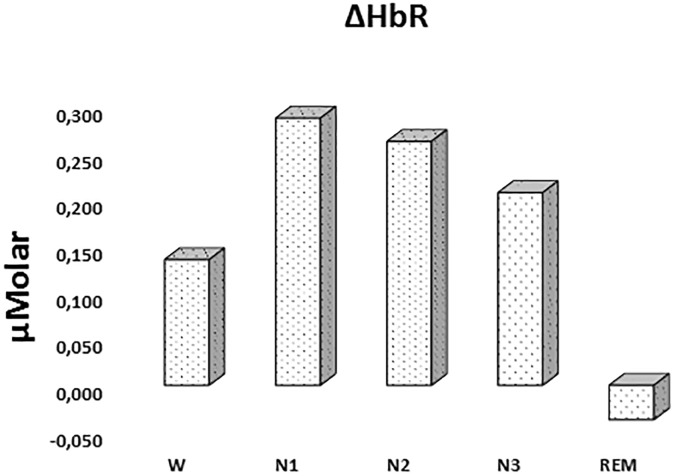
Mean ΔHbR levels for each stage throughout the night. Vertical axis represents deoxyhemoglobin level compared to baseline fNIRS measurement in μMolar. Each bar represents separate sleep stage. W, wakefulness; N1, stage 1; N2, stage 2; N3, stage 3; REM, rapid eye movement stage.

There was a statistically significant difference between ΔHbR levels in different stages as determined by one-way ANOVA [*F*(4,908) = 1.342, *p* = 0.252] (Figure 3 and Table 2).

#### ΔHbT

**FIGURE 4:**
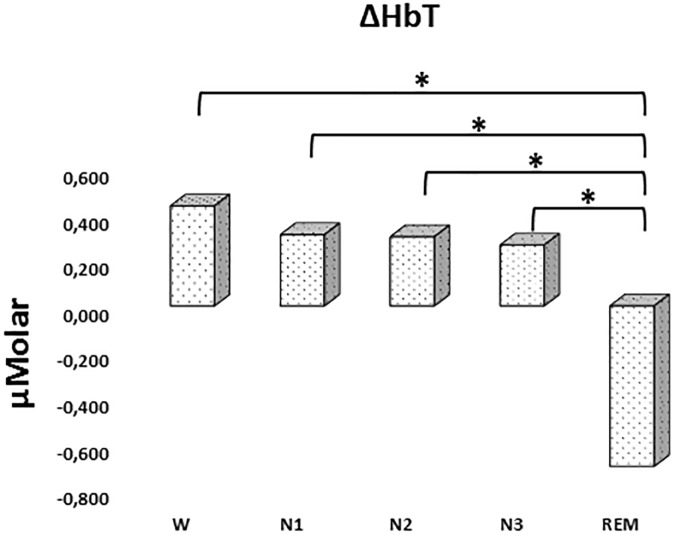
Mean ΔHbT levels for each stage throughout the night. Vertical axis represents total hemoglobin level compared to baseline fNIRS measurement in μMolar. Each bar represents separate sleep stage. W, wakefulness; N1, stage 1; N2, stage 2; N3, stage 3; REM, rapid eye movement stage (significant differences are denoted as follows: ^∗^*p* < 0.05).

There was a statistically significant difference between ΔHbT levels in different stages as determined by one-way ANOVA [*F*(4,906) = 2.942, *p* = 0.020]. A Bonferroni *post hoc* test revealed that ΔHbT was statistically significantly lower in REM (-0.698 ± 0.275) compared to W (0.435 ± 0.158, *p* = 0.012), N1 (0.310 ± 0.143, *p* = 0.018), N2 (0.301 ± 0.128, *p* = 0.013), and N3 (0.265 ± 0.154, *p* = 0.028) (Figure 4). There was no statistically significant difference in other comparisons (*p* > 0.960) (Table 2).

### The Beginning vs. the End of the Sleep

#### ΔHbO

**FIGURE 5:**
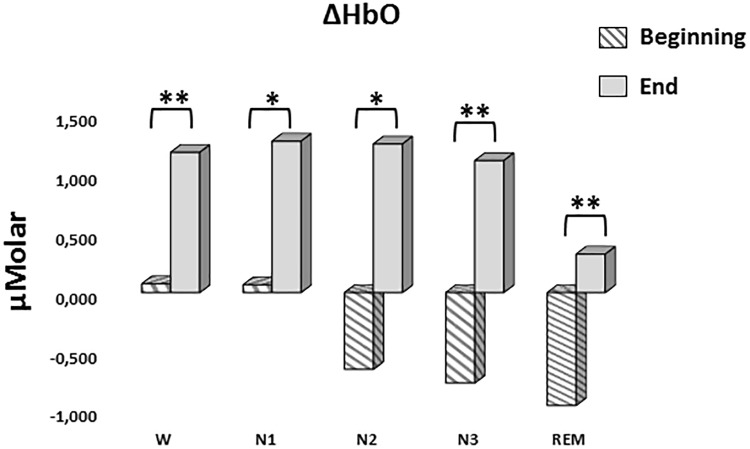
Mean ΔHbO levels for each stage at the beginning and end of sleep. Vertical axis represents oxyhemoglobin level compared to baseline fNIRS measurement in μMolar. Each bar represents separate sleep stage. W, wakefulness; N1, stage 1; N2, stage 2; N3, stage 3; REM, rapid eye movement stage (significant differences are denoted as follows: ^∗^*p* < 0.05, ^∗∗^*p* ≤ 0.01).

A two-way repeated measures ANOVA was conducted to compare the main effects of stage and time and the interaction effect between stage and time on ΔHbO levels. The main effect for time yielded an *F* ratio of *F*(1,7) = 32.539, *p* < 0.001, indicating a significant difference between the ΔHbO levels at the beginning (-0.442 ± 0.165) and the end of sleep (1.027 ± 0.267). There was no statistically significant main effect for stage on ΔHbO levels, *F*(4,28) = 1.794, *p* = 0.158 (Figure 5). There was no statistically significant interaction between the effects of stage and time on ΔHbO levels, *F*(4,28) = 0.752, *p* = 0.565. ΔHbO levels at the beginning and the end of sleep differed for each stage (*p* < 0.05) (Table 3).

**Table 3:** Hemoglobin levels (estimated marginal means ± standard error) compared to baseline recording for each stage at the beginning and end of sleep.

		*ΔHbO (μMolar)*	*ΔHbR (μMolar)*	*ΔHbT (μMolar)*
*W*	*Beginning*	0.074 ± 0.216	0.473 ± 0.218	0.359 ± 0.428
	*End*	1.179 ± 0.403	-0.684 ± 0.194	0.279 ± 0.604
*N1*	*Beginning*	0.065 ± 0.280	0.870 ± 0.304	0.705 ± 0.322
	*End*	1.273 ± 0.488	-0.478 ± 0.342	0.833 ± 0.426
*N2*	*Beginning*	-0.644 ± 0.610	0.909 ± 0.243	0.206 ± 0.721
	*End*	1.250 ± 0.344	-0.575 ± 0.175	0.729 ± 0.430
*N3*	*Beginning*	-0.759 ± 0.349	1.016 ± 0.343	0.123 ± 0.515
	*End*	1.110 ± 0.345	-0.099 ± 0.242	0.634 ± 0.509
*REM*	*Beginning*	-0.948 ± 0.389	0.499 ± 0.366	-0.449 ± 0.591
	*End*	0.323 ± 0.422	-0.821 ± 0.324	-0.499 ± 0.677


*W, wakefulness; N1, stage 1; N2, stage 2; N3, stage 3; REM, rapid eye movement stage.*

#### ΔHbR

**FIGURE 6:**
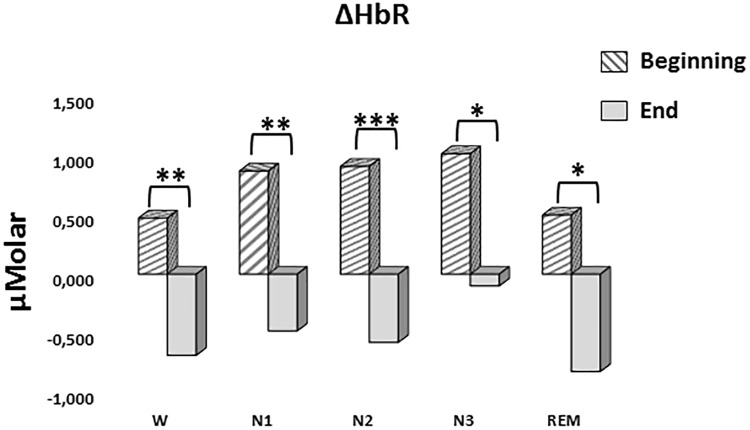
Mean ΔHbR levels for each stage at the beginning and end of sleep. Vertical axis represents deoxyhemoglobin level compared to baseline fNIRS measurement in μMolar. Each bar represents separate sleep stage. W, wakefulness; N1, stage 1; N2, stage 2; N3, stage 3; REM, rapid eye movement stage (significant differences are denoted as follows: ^∗^*p* < 0.05, ^∗∗^*p* ≤ 0.01, ^∗∗∗^*p* ≤ 0.001).

A two-way repeated measures ANOVA was conducted to compare the main effects of stage and time and the interaction effect between stage and time on ΔHbR levels. The main effect for time yielded an *F* ratio of *F*(1,7) = 42.628, *p* < 0.001, indicating a significant difference between the ΔHbR levels at the beginning (0.753 ± 0.188) and the end of sleep (-0.531 ± 0.117) (Figure 6). There was no statistically significant main effect for stage on ΔHbR levels, *F*(4,28) = 1.299, *p* = 0.294. There was no statistically significant interaction between the effects of stage and time on ΔHbR levels, *F*(4,28) = 0.275, *p* = 0.892. ΔHbR levels at the beginning and the end of sleep differed for each stage (*p* < 0.05) (Table 3).

#### ΔHbT

**FIGURE 7:**
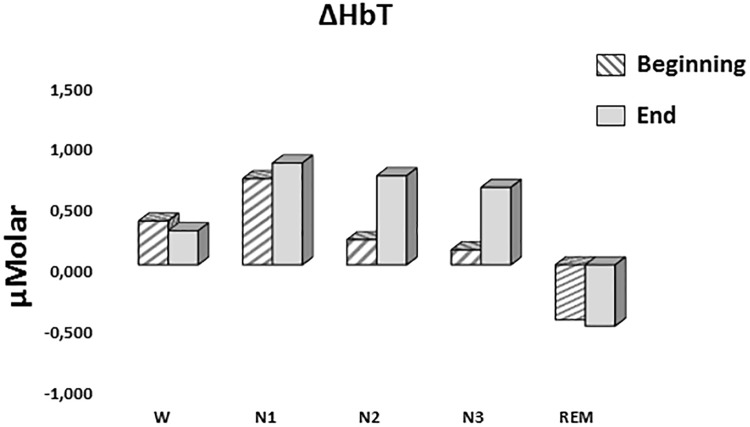
Mean ΔHbT levels for each stage at the beginning and end of sleep. Vertical axis represents total hemoglobin level compared to baseline fNIRS measurement in μMolar. Each bar represents separate sleep stage. W, wakefulness; N1, stage 1; N2, stage 2; N3, stage 3; REM, rapid eye movement stage.

A two-way repeated measures ANOVA was conducted to compare the main effects of stage and time and the interaction effect between stage and time on ΔHbT. There was no statistically significant main effect for time [*F*(1,7) = 0.477, *p* = 0.512] or stage [*F*(4,28) = 1.374, *p* = 0.268] on ΔHbT levels (Figure 7). There was no statistically significant interaction between the effects of stage and time on ΔHbT levels, *F*(4,28) = 0.358, *p* = 0.843 (Table 3).

## Discussion

According to our data, during REM stage of sleep prefrontal cortex has lower levels of oxygenated and total hemoglobin compared to other stages of sleep and wakefulness state whereas deoxygenated hemoglobin level does not differ between these stages. Decreased HbO in REM seems to contradict a study in which ΔHbO increased after onset of REM sleep in daytime sleep (Kubota et al., 2011). This finding of Kubota and colleagues belongs only to the first 30 s of REM sleep not the whole stage. It is possible that HbO level first elevates and then decreases as the REM stage prolongs. Although TOI (HbO/HbT) in REM was higher than stage 2 (Kubota et al., 2011), this could be resulted from decreased HbT rather than increased HbO which was observed in our study. Review on PET studies also shows that rCBF in PFC is decreased in SWS and REM (Maquet, 2000). Decrease in REM can be explained by relative deactivation of PFC rather than absolute. However, the other results of Kubota and colleagues study show that HbO level in whole REM is also higher than stage 2. This contradiction between these studies about HbO and HbT levels may have arisen from the differences of night and daytime sleep recordings. Their subjects were also restricted to sleep 2 h less than their usual in the previous night. Sleep deficiency might also cause differences in sleep hemodynamics. In another daytime sleep study which investigates wakefulness-to-sleep and sleep-to-wakefulness transitions and restricted the subjects’ sleep only up to 4 h in the previous night, HbO, HbR, and HbT decreased during wakefulness-to-sleep transition and they all increased during sleep-to-wakefulness transition (Spielman et al., 2000). HbO and HbR fluctuations desynchronized just before these transitions and are highly synchronized just after the transition.

Further investigations on transition between different sleep stages, and also between sleep and wakefulness revealed that transition to deeper sleep states and from wakefulness to sleep lead to increase in HbR level coupled with decreased SpO2; while the reverse transitions resulted in increased HbO and decreased HbR without a significant change in SpO2 (Näsi et al., 2011). This may indicate that hemodynamics during deepening of sleep mainly involves systemic physiological responses while hemodynamics in transitions toward awakening or from deeper to lighter sleep stages is mainly affected by cerebral activities. In another study investigating transitional changes, researchers inspected stage transitions in adolescents and found that HbR increases during transition from wakefulness and REM to NREM while HbO increases during transition from NREM to REM (Metz et al., 2014). It seems that increased HbO during onset of REM sleep is a consistent finding while the hemodynamics in the overall REM stage results in contradictory findings. This points to distinct mechanisms or quantitative variations in them at the beginning and rest of REM stage.

N3 sleep stage (deep sleep or slow wave sleep in other words) is thought to be the stage where cellular and molecular “cleaning” occurs (Tononi and Cirelli, 2003). However, role of REM in this process also needs to be discussed in the light of our findings. The cleaning mechanisms themselves require energy, but as this cleaning mission is accomplished, energy, hence oxygen requirement of the cells would decrease. So in a short daytime sleep, where only no or few REM stage is observed, brain would have limited time for molecular/cellular cleaning work. This may lead to increased blood flow demand for REM in daytime sleep. But in night sleep, especially toward the end of night, REM stage would have already accomplished refreshing duty and require less blood flow. The reduced HbO level in night sleep would be resulted from this decreased blood supply rather than increased oxygen consumption. This decreased regional blood supply is maintained by increased neuronal activity in other regions of the brain during REM sleep (Maquet, 2000). Low HbT coupled with low HbO in REM in our results supports this idea. So investigating differences in brain hemodynamics between the beginning and end of sleep is crucial.

Our data states that regardless of the sleep stage, oxygenated hemoglobin levels are higher toward the end of whole night sleep than the beginning of it. Deoxygenated hemoglobin levels display a reverse trend resulting in no net change in total hemoglobin levels. According to a study that measured hemoglobin levels both in muscle and cortex during sleep, HbO level decreases and HbR level increases during onset of sleep but this trend is reversed during transition to deep sleep (SWS) (Zhang and Khatami, 2013). As the researchers observed this transition both in brain and muscle tissue, they concluded that it is a systemic physiological response rather than having cortical specificity. Their data seems to reflect higher HbO and lower HbR during the second half of sleep in brain but not in muscle. Unfortunately, they didn’t analyze this issue. A study just got closer to investigate this issue and revealed that HbO after sleep is higher than HbO before sleep, and HbR after sleep is lower than HbR before sleep in adolescents (Metz et al., 2013). They concluded that these results are compatible with synaptic homeostasis hypothesis. Although they recorded brain oxygenation data of whole night sleep and also scored sleep stages, they did not compare stages with each other or the same stages from the beginning and the end of sleep in this article.

Future studies would benefit from hemodynamic data from other regions of the brain, using appropriate fNIRS systems. Calculating and analyzing various hemodynamic parameters other than mean value, i.e., skewness, kurtosis, signal slope, peak number, sum of peaks, peak value, etc. (Khan and Hong, 2015), would provide insight about hemodynamic mechanisms during sleep. Using more parameters would result in multiple comparison problem and would require reduced significance threshold and decreased statistical power in turn. Increasing participant number would be appropriate for enhancing the analysis power.

## Conclusion

During REM sleep, PFC seems to get lower blood supply, possibly due to increased demand in other brain regions. Regardless of the stage, PFC has higher oxygenation toward the end of sleep, indicating a refreshment.

Overall, our brain seems to be on duty during sleep throughout the night for “cleaning” and “refreshing” itself. Hemodynamic changes from the beginning to end of sleep might be the indicator of this work. Thus, accordingly REM stage seems to be at a central point for this work.

## Author Contributions

AO, GI, and MO contributed substantially to the design of the work. GI acquired the data. GI and ST contributed substantially to the analysis of the data. AO, GI, ST, CG, and MO contributed substantially to the interpretation of the data. GI, ST, and CG wrote the first and final drafts of the manuscript. AO and MO contributed substantially to the critical revision of the manuscript.

## Conflict of Interest Statement

The authors declare that the research was conducted in the absence of any commercial or financial relationships that could be construed as a potential conflict of interest.
